# Influence of Social Workers' Empathy Ability on Suicidal Ideation of Cancer Patients

**DOI:** 10.3389/fpubh.2022.925307

**Published:** 2022-07-22

**Authors:** Ningxi Yang, Yuting Zhang, Zhibo Liu, Fang Wang, Guoqing Yang, Xiuying Hu

**Affiliations:** ^1^College of Humanities and Social Sciences, Harbin Engineering University, Harbin, China; ^2^Innovation Center of Nursing Research, Nursing Key Laboratory of Sichuan Province, West China School of Medicine/West China Hospital, Sichuan University, Chengdu, China; ^3^Health Science Center, Shenzhen University, Shenzhen, China

**Keywords:** cancer patient, medical social work, empathy, psychosocial oncology, suicidal ideation

## Abstract

**Background:**

The nursing goal of patients with cancer is to provide them with holistic care, including physical, psychological, and social adaptation, and spirituality. This research aimed to explore the influence of the social workers' empathy ability on suicidal ideation of patients with cancer and its path.

**Methods:**

There was a sum of 358 patients with cancer and the 45 social workers serving them participated in the survey. Data of their self-efficacy, depression symptom, stigma, and suicidal ideation were measured before the social work provided (T1) and 3 months after the social work finished (T2) were collected and compared. Pearson correlation analysis was used to assess the relationships between social workers' empathy ability and patient indicators at T2. The influence path of social workers' empathy ability on cancer patients' suicidal ideation was explored by path analysis at T2.

**Results:**

At T2, patients reported higher self-efficacy and lower depression symptoms, stigma, and suicidal ideation than at T1. At T2, social workers' empathy ability was positively related to patients' self-efficacy and was negatively related to depression symptoms, stigma, and suicidal ideation. Social workers' empathy ability affected patients' suicidal ideation directly. In addition, patients' self-efficacy, depression symptoms, and stigma played mediating roles in the influence of social workers' empathy abilities on their suicidal ideation.

**Conclusion:**

Social workers' empathy ability not only directly affected cancer patients' suicide ideation but also affected suicide ideation through the mediating roles of self-efficacy, depression symptoms, and stigma. Therefore, the improvement of the empathy ability of medical social workers needs to be paid attention to.

## Introduction

Health management of patients with cancer has always been a multidisciplinary issue. It requires not only the participation of doctors and nurses but also psychotherapists and social workers to help patients improve their mental health and social adaptation. In some developed countries in Europe and the United States, medical social workers have become a mature and specialized profession to intervene in the medical process of patients in the whole process, and their education and training system is sound (1). Patients with cancer face the threat of long course of the disease, treatment cycle, and even death, which destroys their mental health and social function (2, 3). Therefore, they need social work services as part of holistic care and health management. Oncology social worker has become an independent profession in countries, such as Sweden (4). In mainland China, although medical social work started in Peking Union Medical College Hospital as early as 1921, the development and the accessibility of medical social work are limited even in the areas with the fastest development of social work, such as Guangdong Province (5, 6). Beijing is one of the cities with the fastest development of medical social work in China. However, by the end of 2021, there were 265 medical social workers in Beijing, including 113 full-time medical social workers and 152 part-time medical social workers (7). And now in some medical schools or colleges of social sciences in comprehensive universities, medical social work is one of the training directions of the master of the social work program. However, the number of students in this direction is limited (8). In 2015, the Chinese government proposed the Healthy China Strategy, which provided a new opportunity for the development of medical social work (9). In this context, we carried out this study, hoping to explore the role of medical social work on patient health through empirical research.

Empathy refers to the ability to experience the situation of others, to feel and understand the feelings of others (10). Previous studies have confirmed the impact of medical staff's empathy on patients' outcomes (11). Researchers believed that medical staff with high empathy ability were of great benefit to patients' physical and mental health (12, 13). In addition, in medical school, the cultivation and assessment of students' empathy ability have become the norm (14). This shows that medical educators have realized the importance of the empathy ability of medical staff. Similarly, medical social workers need close contact with patients and psychosocial support, so their empathy ability is also very important.

However, in medical practice, especially from the perspective of psychosocial oncology, the impact of social workers' empathy on patients' psychosocial health has not been reported. According to previous research and clinical practice, patients with cancer are prone to psychosocial problems, such as suicidal ideation, stigma, depression symptom, anxiety symptom, insufficient self-efficacy, and sleep disorder (15, 16). Therefore, they need medical social workers to empower them through professional services.

According to the literature, self-efficacy is a protective factor against suicidal ideation, and increasing self-efficacy can be strategically beneficial for larger suicide prevention (17). To some extent, the self-efficacy of patients with cancer is an instance mental health indicator that is closely related to self-health management (18). On the contrary, it has been confirmed that depressive symptoms and stigma are risk factors for suicidal ideation (19).

For patients with cancer, many people have depressive symptoms and stigma because of the severity of the disease, the negative impact on the patient's physical function and social function, and the stigmatization of the disease (20, 21). Therefore, reducing risk factors and increasing protective factors are the focus of suicide ideation prevention. Social work service is an important way to improve the psychological and social health of patients with cancer. As the dominator of social work service, medical social workers provide psych-social support to the patients and their empathy ability is closely related to service quality. Some social work services themselves are carried out through communication and empathy between social workers and patients. As mentioned above, the influence of the empathy ability of the medical staff in contact with the patient on the physical and mental health indicators of the patient has been confirmed (11, 12). This provides a basis for social workers' empathy ability to have an impact on patients. The above constitutes the theoretical basis of this article: In the social work service, social workers' empathy ability affected the suicidal ideation of patients with cancer by influencing the protective factors and risk factors of suicidal ideation.

Based on this background, this study explores the impact of empathy of social workers on suicidal ideation of patients with cancer and tries to analyze the action path. Through this study, we hope to verify the impact of the empathy ability of social workers on patients' psychosocial health and call on medical social workers to pay attention to the improvement of their empathy ability. We established two hypotheses about the impact of social workers' empathy on suicidal ideation in patients with cancer. Hypothesis 1: Social workers' empathy ability affected cancer patients' suicidal ideation directly. Hypothesis 2: Social workers' empathy ability affected cancer patients' suicidal ideation through the mediating roles of self-efficacy, depression symptoms, and stigma.

## Materials and Methods

### Samples

From 2019 to 2021, 358 patients with cancer were selected as the research objects by using the convenient sampling method and were asked to complete the questionnaires. Because the total number of medical social workers in mainland China is very small, these social workers and patients come from Beijing, Guangdong, and other provinces. The inclusion criteria of patients: (1) They were diagnosed with cancer and knew their diagnosis. (2) They had no mental illness and did not use psychotropic drugs. (3) They would receive social work case service. Social workers serving them were also asked to fill out questionnaires. Each social worker can serve multiple patients, but each patient can only receive the services of a single social worker.

The inclusion criteria of the medical social worker: (1) Since there is no medical social worker certificate issued by a special authority in mainland China, participants are required to hold a social worker certificate. (2) They should actually engage in medical social work for more than 6 months after obtaining the social work qualification. (3) They should be social workers from hospitals, social work agencies, or NGOs.

### Procedures

This research aimed to confirm whether there was a relationship between social workers' empathy ability and patients' suicide ideation. We performed a mediation analysis to test how social workers' empathy ability affected patients' suicide ideation. The main steps of the research were as follows: (1) Before the social work provided (T1), patients were asked to complete an anonymous questionnaire, including questions on demographic sociological information and disease situation. And their self-efficacy, depression symptoms, stigma, and suicide ideation were measured. Their social workers' empathy abilities were measured. Because empathy is a relatively stable ability, we only measured it once. (2) According to unified standards, social workers provided case services for patients, including helping patients and families fully understand their own resources and potential, psychosocial health counseling, improving their self-management and coping abilities, and improving their abilities to adapt to the treatment stage and solve difficulties. The social work was provided at least 6 times for each patient, and each time lasted for 30–60 min. These six services were completed within 2 months. The services were provided in hospitals or in patients' homes according to the actual situation. (3) Three months after the social work finished (T2), patients' psychological indicators were measured and were compared with T1. The relationships between social workers' empathy ability and patient indicators at T2 were measured. The influence of social workers' empathy ability on cancer patients' suicidal ideation and its path was explored.

### Measures

#### Essential Information

The data of demographic information of patients comprised sex, age, family residence (countryside/county town/urban area), and disease situation was collected.

#### Interpersonal Reactivity Index (IRI)

The IRI questionnaire was applied to evaluate social workers' empathy ability. The IRI consists of a 5-point Likert Scale with 22 items. These items are divided into four dimensions: Perspective taking, empathic concern, fantasy, and personal distress. The score for each item ranges from 0 to 4, and the total score ranges from 0 to 88. A higher score indicates a stronger empathy ability (22). The Cronbach's alpha of the scale in this research was 0.8.

#### Cancer Behavior Inventory (CBI)

The CBI questionnaire was applied to measure patients' self-efficacy. The CBI consists of 12 items. These items are divided into four dimensions: maintaining independence and a positive attitude, participating in medical care, coping and stress management, and managing effect. The score for each item ranges from 1 to 9, and the total score ranges from 12 to 108. A higher score indicates a stronger self-efficacy (23). The Cronbach's alpha of the scale in this research was 0.85.

#### Self-Rating Depression Scale (SDS)

The SDS questionnaire was applied to assess patients' depression symptoms. Patients were asked to use their last week's mental state as a reference when answering the survey questions. The SDS consists of a 4-point Likert Scale with 20 questions (10 positive statements and 10 negative statements). The score for each item ranges from 1 to 4, and the total score ranges from 20 to 80. The standard score is the total score multiplied by 1.25. A higher score indicates more severe depression symptoms (24). The Cronbach's alpha of the scale in this research was 0.82.

#### Social Impact Scale (SIS)

The SIS questionnaire was applied to measure patients' stigma. The SIS consists of a 4-point Likert Scale with 24 items. These items are divided into four dimensions: social rejection, financial insecurity, internalized shame, and social isolation. The score for each item ranges from 1 to 4, and the total score ranges from 24 to 96. A higher score indicates more stigma (25). The Cronbach's alpha of the scale in this research was 0.85.

#### Beck Scale for Suicide Ideation (BSI)

The BSI questionnaire was applied to test patients' suicide ideation. The BSI consists of 19 items. The score for each item ranges from 0 to 2, and the total score ranges from 0 to 38. A higher score indicates more suicide ideation (26). The scale has been validated in patients with cancer in former studies (27, 28). The Cronbach's alpha of the scale in this research was 0.81.

### Statistical Analysis

EpiData Entry version 3.1 and SPSS version 25.0 was applied to enter and analyze the data. The data were tested for normality with a P-P diagram. In this research, all data was presented by mean ± SD because it conformed to the normal distribution. The paired *t*-test was applied to compare the indicators on T1 and T2. After the data were proved to be consistent with the homogeneity of variance, variance analysis was applied to compare the indicators of the groups. Pearson correlation analysis was used to test the correlations between social workers' empathy ability and patients' indicators. Structural equation modeling (SEM) was applied to analyze the relationships between variables. SPSS Amos version 25.0 was applied to assess variables' mediation effects. Setting the bootstrap number as 5,000, the bias-corrected non-parametric percentile bootstrap method was used to estimate the significance of specific mediating effects and simple slope analysis. A *P* value < 0.05 was considered statistically significant.

## Results

### Samples

There were a total of 380 patients were asked to participate in the research, and 358 of them provided valid questionnaires, with a participation rate of 94.21%. Their average age was (55.2 ± 3.8) years. Gender distribution: 170 were male, accounting for 47.49%; 188 were female, accounting for 52.51%. Family residence distribution: 127 came from rural areas, accounting for 35.47%; 31 came from county towns, accounting for 8.66%; and 200 came from urban areas, accounting for 55.87%. Their average course of disease was (4.2 ± 0.8) years. There were 78 patients had respiratory cancer (lung cancer, nasopharyngeal carcinoma, laryngocarcinoma, and bronchial gland tumor), 85 patients had gastrointestinal cancer (liver, stomach, colorectal, or esophagus cancer), 62 patients had reproductive system cancer (cervical, ovarian, prostate, testicular, or penis cancer), 23 patients had urinary system cancer (kidney, renal pelvis, ureter, bladder, or urethra cancer), 68 patients had breast cancer, and 42 patients had head and neck neoplasms. The 358 patients with cancer were served by 45 social workers, with an average service time of 278 min in total for each patient.

### Comparison of Psychological Indexes of Patients Before (T1) and 3 Months After (T2) Social Work Service

The psychological indexes of the patients at T1 and T2 were compared. At these two time points, the psychological indicators of patients showed significant differences. The results are shown in Table 1.

**Table 1:** Comparison of psychological indexes of patients before (T1) and 3 months after (T2) social work service.

	**T1**	**T2**	***t***	***P***
CBI	67.134 ± 14.624	74.827 ± 17.338	23.752	<0.0001
SDS	58.277 ± 9.254	51.662 ± 12.939	20.272	<0.0001
SIS	57.73 ± 9.645	54.391 ± 11.802	4.074	<0.0001
BSI	12.405 ± 7.435	9.413 ± 7.968	5.194	<0.0001

To clarify the situation of patients with different types of cancer, we compared the psychological indexes in 6 types of patients with cancer at T2. There was a significant difference in the comparison of stigma (SIS) among the groups. Patients with breast cancer had the highest stigma. The results are shown in Table 2.

**Table 2:** Comparison of psychological indexes of patients with different types of cancer.

	**Respiratory cancer (*n* = 78)**	**Gastrointestinal cancer (*n* = 85)**	**Reproductive system cancer (*n* = 62)**	**Urinary system cancer (*n* = 23)**	**Breast cancer (*n* = 68)**	**Head and neck cancer (*n* = 42)**	***F***	***P***
CBI	75.15 ± 16.65	73.53 ± 18.33	76.37 ± 16.91	72.78 ± 19.81	74.79 ± 16.42	75.74 ± 17.92	0.283	0.922
SDS	52.56 ± 12.90	50.65 ± 13.92	51.21 ± 13.15	51 ± 11.95	53.57 ± 12.47	49.98 ± 12.15	0.644	0.666
SIS	53.04 ± 10.79	53.95 ± 12.60	55.35 ± 11.98	54.70 ± 11.59	57.82 ± 12.05	50.64 ± 10.32	2.356	0.040
BSI	8.41 ± 7.09	9.74 ± 7.72	7.94 ± 7.19	10.91 ± 9.51	11.16 ± 9.29	9.14 ± 7.61	1.542	0.176

### Correlations Among Social Workers' Empathy Ability (IRI), Patients' Self-Efficacy (CBI), Depression Symptoms (SDS), Stigma (SIS), and Suicide Ideation (BSI) at T2

At T2, social workers' empathy ability was positively correlated with patients' self-efficacy, and negatively correlated with patients' self-efficacy, depression symptoms, stigma, and suicidal ideation. Table 3 shows the results at T2 in detail.

**Table 3:** Correlations among social workers' empathy ability (IRI), patients' self-efficacy (CBI), depression symptoms (SDS), stigma (SIS), and suicide ideation (BSI) at T2.

	**Mean**	**SD**	**IRI**	**CBI**	**SDS**	**SIS**	**BSI**
IRI	57.159	13.123	1				
CBI	74.827	17.338	0.426^**^	1			
SDS	51.662	12.939	−0.414^**^	−0.449^**^	1		
SIS	54.391	11.802	−0.396^**^	−0.299^**^	0.363^**^	1	
BSI	9.413	7.968	−0.771^**^	−0.494^**^	0.466^**^	0.448^**^	1

** *P < 0.01*.

### Path Analysis of the Effects of Social Workers' Empathy Ability (IRI) on Patients' Suicide Ideation (BSI) at T2

**Figure 1:**
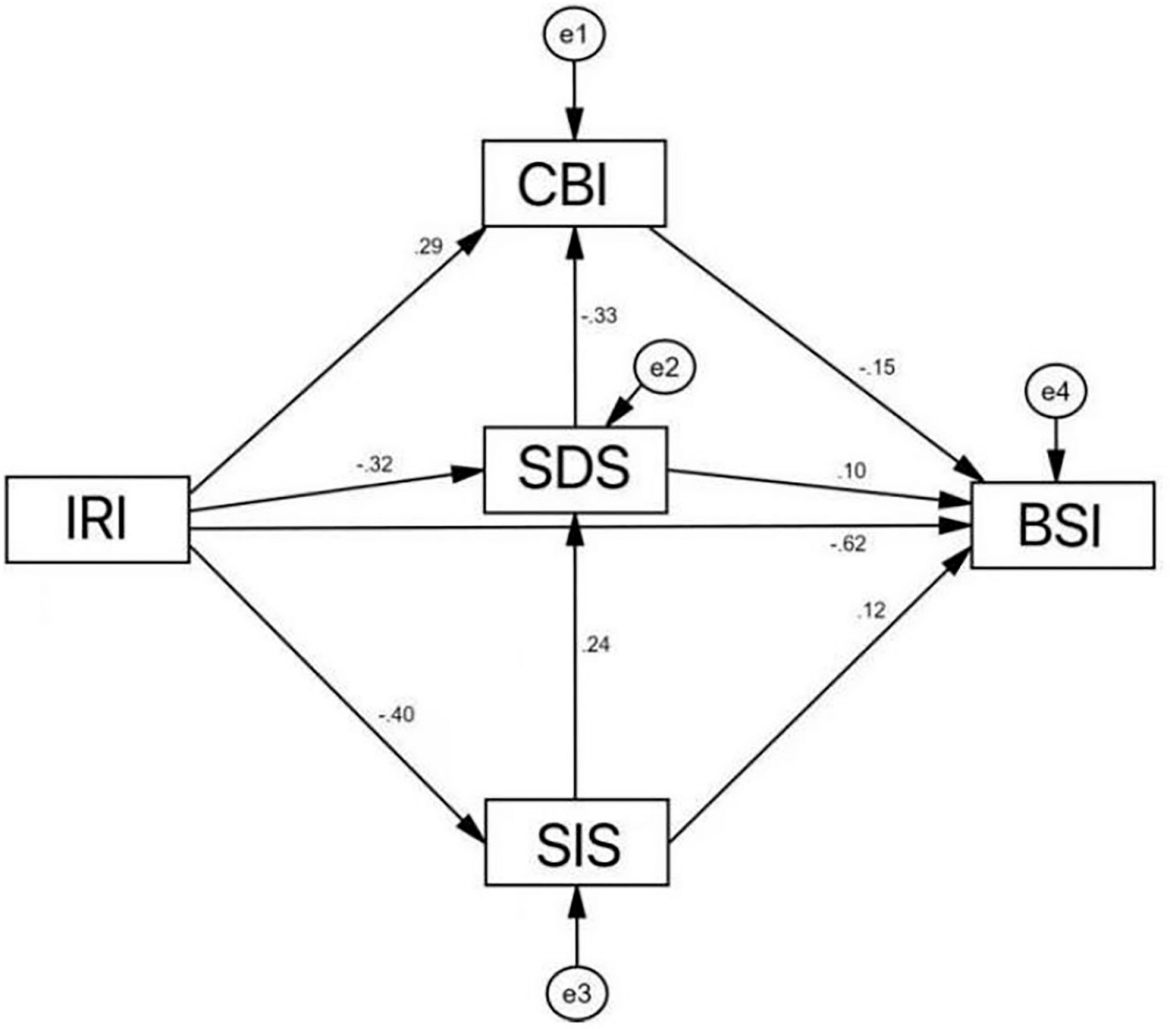
The model of relationships among IRI, CBI, SDS, and BSI, with standardlized beta weights.

The results of path analysis showed that social workers' empathy ability had a significant negative impact on patients' suicide ideation (*P* < 0.001), which meant that social workers' empathy ability affected patients' suicide ideation directly. Therefore, hypothesis 1 was confirmed. Moreover, social workers' empathy ability had a significant positive impact on patients' self-efficacy and a significant negative impact on patients' depression symptoms and stigma. Patients' self-efficacy had a significant negative impact on their suicide ideation. And patients' depression symptoms and stigma had a significant positive impact on their suicide ideation. Table 4 shows the results in detail. The fitting index of structural equation model was ideal: χ^2^/*df* = 2.615, RMSEA = 0.067, GFI = 0.997, TLI = 0.974, CFI = 0.997, IFI = 0.997, AGFI = 0.956, and NFI = 0.996. The mediating effect model is shown in Figure 1.

**Table 4:** Path analysis of the effects of social workers' empathy ability (IRI) on patients' suicide ideation (BSI) at T2.

**Path**			**Standardization coefficient**	**Unstandardized coefficient**	**S.E**.	**C.R**.	***P***
IRI	→	SIS	−0.396	−0.356	0.044	−8.154	^***^
IRI	→	SDS	−0.320	−0.316	0.050	−6.283	^***^
SIS	→	SDS	0.236	0.258	0.056	4.625	^***^
IRI	→	CBI	0.289	0.382	0.066	5.826	^***^
SDS	→	CBI	−0.330	−0.442	0.066	−6.648	^***^
SDS	→	BSI	0.099	0.061	0.023	2.638	0.008
SIS	→	BSI	0.122	0.083	0.024	3.486	^***^
IRI	→	BSI	−0.619	−0.375	0.023	−16.510	^***^
CBI	→	BSI	−0.150	−0.069	0.017	−4.093	^***^

*** *P < 0.001*.

### Mediation Effect of Patients' Self-Efficacy (CBI), Depression Symptoms (SDS), and Stigma (SIS) in the Effect of Social Workers' Empathy Ability (IRI) on Patients' Suicide Ideation (BSI) at T2

Table 5 presents the results of Bootstrap indirect influence analysis. When the CI does not include 0, the following results will be achieved. In the path of IRI → SDS → BSI, SDS played a significant mediating effect between IRI and BSI (β = −0.032, *P* < 0.01). In the path of IRI → CBI → BSI, CBI played a significant mediating effect between IRI and BSI (β = −0.043, *P* < 0.001). In the path of IRI → SIS → BSI, SIS played a significant mediating effect between IRI and BSI (β = −0.048, *P* < 0.001). In the path of IRI → SDS → CBI → BSI, SDS, and CBI had a significant chain-mediating effect between IRI and BSI (β = −0.016, *P* < 0.001). In the path of IRI → SIS → SDS → BSI, SIS, and SDS had a significant chain mediating effect between IRI and BSI (β = −0.009, *P* < 0.01). In the path of IRI → SIS → SDS → CBI → BSI, SIS, SDS, and CBI had a significant chain-mediating effect between IRI and BSI (β = −0.005, *P* < 0.001). Therefore, hypothesis 2 was confirmed.

**Table 5:** Mediation effect of patients' self-efficacy (CBI), depression symptoms (SDS), and stigma (SIS) in the effect of social workers' empathy ability (IRI) on patients' suicide ideation (BSI) at T2.

**Path of intermediary effect**	**Standardization coefficient**	**Unstandardized coefficient**	**S.E**.	**95% CI**		***P***
				**Lower**	**Upper**	
IRI → SDS → BSI	−0.032	−0.019	0.011	−0.056	−0.013	0.001
IRI → CBI → BSI	−0.043	−0.026	0.015	−0.077	−0.018	0.000
IRI → SIS → BSI	−0.048	−0.029	0.012	−0.074	−0.027	0.000
IRI → SDS → CBI → BSI	−0.016	−0.010	0.006	−0.032	−0.006	0.000
IRI → SIS → SDS → BSI	−0.009	−0.006	0.004	−0.019	−0.004	0.001
IRI → SIS → SDS → CBI → BSI	−0.005	−0.003	0.002	−0.010	−0.002	0.000

## Discussion

Overall, it was confirmed that patients' self-efficacy increased, and depression, stigma, and suicidal ideation decreased 3 months after the social work service in this study. This showed that social work services were helpful to the mental health of patients with cancer. Among these patients with cancer, breast patients with cancer had the highest stigma. This shows that in the future psychosocial intervention, we should pay more attention to helping them reduce the stigma.

In the path analysis of social workers' empathy ability on patients' suicidal ideation, the two hypotheses in the study have been confirmed: Social workers' empathy ability not only directly affected cancer patients' suicidal ideation but also affected the suicidal ideation of patients by acting on self-efficacy, depression symptoms, and stigma.

Our results showed social workers' empathy ability affected cancer patients' suicidal ideation directly. This just echoes the “offering empathy without endorsing suicidal ideation” advocated in other studies (29). We got this result may be because in the case service, high empathy might help them accurately identify the patients' suicidal ideation and conduct targeted counseling. Even if the psychological support that social workers could give patients was limited, once they timely identified the patients' suicidal ideation, they would invite professionals to intervene with the patients, which could reduce the patients' suicidal ideation.

In addition, social workers' empathy ability affected cancer patients' suicidal ideation indirectly, and self-efficacy, depression symptoms, and stigma were mediating roles. In the first path, social workers' empathy ability affected patients' suicidal ideation through the mediating role of self-efficacy. Although there haven't been reported the effect of social workers' empathy ability on patients' psychological indicators, the results were similar to the studies on medical staff and patients. It has been proved doctors' empathy ability was associated with patients' self-efficacy (30). In social work case services for patients with cancer, social workers need to undertake the following tasks (31–33): Promote patients to adapt to the hospital environment in time, and assist in solving their difficulties and problems. Investigate and evaluate the psychosocial health status of patients, appease and alleviate the emotional problems of patients and their families, stimulate their self-efficacy, and improve their ability to use their own and relevant resources to solve problems. Carry out health education and health management for patients to help improve their quality of life. Help patients understand relevant policies, help solve economic problems and seek relevant support. Provide hospice care for end-stage patients. Help coordinate doctor-patient relationships. In the above links, social workers with high empathy ability could fully communicate with patients and understand, respect, and respond to their narration, which was similar to the concept of narrative medicine practiced by medical staff (34, 35). Based on fully understanding the situation of patients, social workers provided targeted and individualized services to help them establish a more positive outlook on suffering, life and death, and medical treatment. In addition, social workers had in-depth exchanges with patients' families and medical staff to discuss how to better help and understand patients, which could increase social support and resources for patients (36, 37). Therefore, patients could feel more support, warmth, and love, which helped them improve their self-efficacy (38). Published works have confirmed that patients with low self-efficacy had more suicidal ideation (39). When patients have higher self-efficacy, they will do better in self-health management, respond to diseases with a more positive and optimistic attitude, and then reduce suicidal ideation. When patients' self-efficacy was low, they could not do their own health management well, and felt out of control, had little faith in survival and self-regulation, and were unwilling to respond to treatment and doctors with a positive attitude (40, 41). These made them immersed in a pessimistic situation and produce suicidal thoughts.

In the second path, social workers' empathy ability affected patients' suicidal ideation through the mediating role of depression symptoms. Social workers with high empathy ability may help reduce patients' depression symptoms. A former study reported that showing perceived physician empathy was related to patients' psychological distress, which was similar to our research (42). When talking with social workers with high empathy ability, patients have more narrative desire. Social workers were also more likely to provide psychological counseling to patients in the narrative and helped patients reconstruct a positive life narrative (43, 44). High empathy helped social workers better complete these processes. These helped to alleviate the depressive symptoms of patients. The depressive symptoms were closely related to suicidal ideation, which has become a consensus (45). People in depressive states tend to think more negatively and may feel more negatively about the meaning of life. They often feel lonely and have low self-evaluation, have no interest in the things of life, and even have suicidal ideation (46).

In the third path, social workers' empathy ability affected patients' suicidal ideation through the mediating role of stigma. A former study confirmed doctors who had strong empathy ability and skills can reduce cancer patients' stigma (47). This is also applicable to social workers. Social workers with high empathy ability can understand the feelings of patients, such as the stigma of patients with breast cancer after mastectomy. People with a strong sense of stigma may have more suicidal ideation. In public opinion, it is often the patient's own reason that leads to cancer, which is also one of the main reasons for the stigma of cancer patients (48). Under the Chinese cultural background, patients or others may feel that people suffer from cancer because they have done bad things. In other words, suffering from cancer is retribution. In addition, suffering from cancer and undergoing surgery may affect their physical appearance and function, interrupt their daily work and life, and bring economic burden and care burden to the family. Therefore, some people have a high sense of stigma. With the aggravation of stigma, suicidal ideation occurs. Therefore, social workers should deepen their understanding of patients' through empathy and help them reduce their stigma. They should help the patients achieve more benefits finding, help them build a correct understanding of the disease and a positive attitude toward the disease, which can reduce their suicidal ideation.

In addition, this study found that the depressive symptoms of patients affected their self-efficacy and stigma. Therefore, there were three chain mediating effects in the influence of social workers' empathy on patients' suicidal ideation.

Although this study had some limitations, such as it was not a big data statistical analysis, and the influencing factors of patients' suicidal ideation were multiple, we could not remove all confounding factors. Because the total number of medical social workers in mainland China is small, it was difficult for us to select social workers in the same region, and it was also difficult to strictly limit the course and type of disease of patients. In our study, the average course of the disease was as long as 4.2 years. In the future, we can carry out some studies on social work services for patients at the initial stage of diagnosis or at the stage of hospice palliative care. We think that social work services may have a better effect on patients at the initial stage of diagnosis, because patients at this stage have just been diagnosed with cancer, which is a major traumatic event for them. Therefore, their psychosocial health needs more attention. In addition, targeted social work services for patients at different disease stages are needed in the future.

The original intention of this study was to call for the involvement of social workers in the health management of cancer patients. And we advocate strengthening empathy training for medical social workers, such as narrative medical education. It is necessary to strengthen the communication between medical staff and social workers and establish a holistic healthcare team to comprehensively improve the physical-mental-social health of patients with cancer.

## Conclusion

In cancer patients, social workers' empathy ability not only affected their suicidal ideation directly but also affected their suicidal ideation through the mediating roles of self-efficacy, depression symptoms, and stigma. Therefore, in terms of industry access to medical social workers, higher requirements for empathy ability should be required. For medical social workers, professionals should provide them with systematic training on humanistic competence, such as empathy.

## Data Availability Statement

The raw data supporting the conclusions of this article will be made available by the authors, without undue reservation.

## Ethics Statement

The studies involving human participants were reviewed and approved by College of Humanities and Social Sciences, Harbin Engineering University. The patients/participants provided their written informed consent to participate in this study.

## Author Contributions

NY and GY: conceptualization. NY: methodology, investigation, resources, and data curation. ZL: software. ZL and FW: validation. YZ: formal analysis. NY and YZ: writing—original draft preparation and writing—review and editing. FW: visualization. GY: supervision. GY and XH: project administration and funding acquisition. All authors have read and agreed to the published version of the manuscript.

## Funding

This study was supported by the Foundation of Nursing Key Laboratory of Sichuan Province (Grant No. HLKF2022-6), Fundamental Research Funds for the Central Universities of Harbin Engineering University (3072022WK1306), SZU Top Ranking Project (86000000210) and 2021 Heilongjiang Higher Education Teaching Reform Research Project (SJGY20210232).

## Conflict of Interest

The authors declare that the research was conducted in the absence of any commercial or financial relationships that could be construed as a potential conflict of interest.

## Publisher's Note

All claims expressed in this article are solely those of the authors and do not necessarily represent those of their affiliated organizations, or those of the publisher, the editors and the reviewers. Any product that may be evaluated in this article, or claim that may be made by its manufacturer, is not guaranteed or endorsed by the publisher.
